# Genetic architecture of glucosinolate variation in *Brassica napus*

**DOI:** 10.1016/j.jplph.2019.06.001

**Published:** 2019-09

**Authors:** Varanya Kittipol, Zhesi He, Lihong Wang, Tim Doheny-Adams, Swen Langer, Ian Bancroft

**Affiliations:** Department of Biology, University of York, Heslington, York, YO10 5DD, UK

**Keywords:** Glucosinolates, Brassica napus, HAG1, HAG3

## Abstract

The diverse biological activities of glucosinolate (GSL) hydrolysis products play significant biological and economical roles in the defense system and nutritional qualities of *Brassica napus* (oilseed rape). Yet, genomic-based study of the *B. napus* GSL regulatory mechanisms are scarce due to the complexity of working with polyploid species. To address these challenges, we used transcriptome-based GWAS approach, Associative Transcriptomics (AT), across a diversity panel of 288 *B. napus* genotypes to uncover the underlying genetic basis controlling quantitative variation of GSLs in *B. napus* vegetative tissues. Single nucleotide polymorphism (SNP) markers and gene expression markers (GEMs) associations identify orthologues of *MYB28/HAG1* (AT5G61420), specifically the copies on chromosome A9 and C2, to be the key regulators of aliphatic GSL variation in leaves. We show that the positive correlation observed between aliphatic GSLs in seed and leaf is due to the amount synthesized, as controlled by *Bna.HAG1.A9 and Bna.HAG1.C2*, rather than by variation in the transport processes. In addition, AT and differential expression analysis in root tissues implicate an orthologue of *MYB29/HAG3* (AT5G07690)*, Bna.HAG3.A3*, as controlling root aromatic GSL variation. Based on the root expression data we also propose *Bna.MAM3.A3* to have a role in controlling phenylalanine chain elongation for aromatic GSL biosynthesis. This work uncovers a regulator of homophenylalanine-derived aromatic GSLs and implicates the shared biosynthetic pathways between aliphatic and aromatic GSLs.

## 1 Introduction

Glucosinolates (GSLs) are a group of sulfur- and nitrogen-rich secondary metabolites prevalent in Brassicales (Halkier and Gershenzon, 2006). GSLs are economically significant because their bioactive hydrolysates have diverse biological properties that impact agriculturally important *Brassica* crops such as oilseed rape (*Brassica napus* L.) and have been studied extensively in the model plant *Arabidopsis thaliana*. Depending on the reaction conditions and GSL side-chain structure, bioactive hydrolysates such as isothiocyanates, nitriles and oxazolidine-2-thione are produced when myrosinase enzymes came into contact with GSLs after tissue damage (Rask et al., 2000; Wittstock and Halkier, 2002). Some GSLs and their hydrolysis products are thought to defend the plants against non-adapted pathogen and insect pests (Glen et al., 1990; Potter et al., 2000; Hopkins et al., 2009), while other isothiocyanates are suitable as biofumigants to control soil pests and weeds (Gimsing and Kirkegaard, 2009). However, other GSLs have negative impacts. For example, progoitrin can accumulate to high concentrations in seeds. When these are hydrolyzed, it produces goitrogenic products that reduce the nutritional values of the protein-rich seed meal used as livestock feed (Griffiths et al., 1998; Tayo et al., 2012). To allow the use of seed meal as animal feed, extensive breeding efforts have been made to select for oilseed rape cultivars with low seed GSLs (<30 μmol/g) (Rosa et al., 1997). On the other hand, the introduction of ‘00′ (low seed erucic and GSL) cultivars, has led to the concern that these cultivars could be more susceptible to pests and diseases due to reduction of the presumed defensive role of GSL. Nevertheless, levels of GSLs and their interaction with plant pests may be more intricate than previously thought because the same GSL profile can acts as both deterrent to generalist pests and stimulant to specialist pests (Mithen, 1992; Giamoustaris and Mithen, 1995; Hopkins et al., 2009). Some studies have reported no significant correlation of GSL between seeds and leaves, suggesting that modifying the GSL profiles selectively in different parts of the plant may be feasible (Porter et al., 1991; Fieldsen and Milford, 1994). However, the underlying genetic control of quantitative variations of GSL in vegetative tissues and seeds of *B. napus*, and their interaction, are not well understood.

**Fig. 1:**
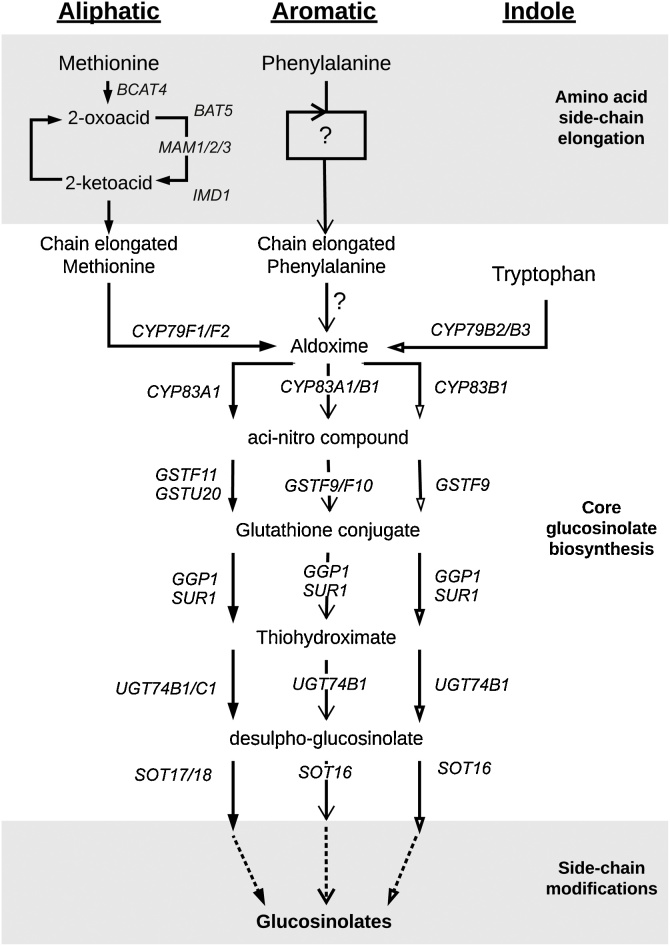
Simplified aliphatic, aromatic and indole glucosinolate biosynthesis pathways in Brassicaceae, comprising of three stages: amino acid side chain elongation, core moiety biosynthesis and extensive side chain modifications. The enzymes involving in phenylalanine chain elongation and catalyzing the subsequent homophenylalanine are unknown.

Based on their amino acid precursor of the side chain, GSLs are divided into three structural groups: aliphatic, indole and aromatic GSLs, which derived from methionine, tryptophan and phenylalanine respectively (Fahey et al., 2001). The biosynthetic pathway of GSLs proceeds in three stages via (i) amino acid side chain elongation; (ii) the amino acid moiety undergoing metabolic configurations to form the core GSL structure; and (iii) secondary modifications of the side chain to generate a wide spectrum of GSL compounds (Fig. 1). Many of the genes responsible for biosynthetic steps have been identified in *Arabidopsis thaliana* (reviewed in Grubb and Abel, 2006; Halkier and Gershenzon, 2006; Sønderby et al., 2010), which has also helped clarify the core biosynthesis steps and identify orthologous genes in the closely related *Brassica* species. A group of R2R3 MYB transcription factors from a single gene family within *Arabidopsis* is known to be involved in the direct transcriptional regulation of GSLs biosynthesis. *MYB34/ATR1*, *MYB51/HIG1*, and *MYB122/HIG2* are thought to regulate the tryptophan-derived indole GSL pathway (Celenza, 2005; Gigolashvili et al., 2007a; Frerigmann and Gigolashvili, 2014), and *MYB28/HAG1, MYB29/HAG3* and *MYB76/HAG2* regulate the methionine-derived aliphatic GSL biosynthetic genes (Gigolashvili et al., 2007b; Hirai et al., 2007; Gigolashvili et al., 2008; Sonderby et al., 2010). Since methionine-derived aliphatic and tryptophan-derived indole GSLs are the two main classes of GSLs found in *A. thaliana* (Brown et al., 2003), significant progress has been made in understanding the biochemistry and the regulatory controls of these two classes of GSLs. However, less information is available for the chain-elongated homophenylalanine-derived aromatic GSL, which is abundant in *Brassica* species (Bhandari et al., 2015). So far, the genes involved in the side chain elongation and the regulatory genes controlling aromatic GSL biosynthesis remain largely uncharacterized. Furthermore, the CYP79A2 that catalyzes phenylalanine substrates has been shown unable to metabolize homophenylalanine into aldoxime (Wittstock and Halkier, 2000), suggesting the enzyme that controls the flux into the biosynthetic pathway of homophenylalanine-derived aromatic GSLs in *B. napus* is yet to be identified.

While some of the natural variation in GSL profiles can be explained by allelic variation of key biosynthetic genes, other differences are likely to be caused by the activity of regulatory loci (Kliebenstein et al., 2001a). Genome-wide association studies (GWAS) provides a powerful method of using genetically diverse population to identify quantitative trait loci (QTLs) at higher resolution by exploiting historical recombination between molecular markers and loci associated with trait variation (Zhu et al., 2008). With the focus on seed quality traits, association studies had been effectively applied to identify clusters of single-nucleotide polymorphisms (SNPs) highly associated with seed GSL content in *B. napus* in recent years (Li et al., 2014; Lu et al., 2014; Gajardo et al., 2015). Nevertheless, to get better understanding of the modular genetic system that regulates GSL natural variations in *B. napus* as a whole, more work is needed to investigate the regulations of GSL in the vegetative tissues and how these variations relate to the GSL profiles in the seed.

In this study we aimed to elucidate the genetic control of GSL biosynthesis in leaves and roots of *B. napus*. We took the approach of firstly undertaking a transcriptome-based GWAS approach. Such a genomics approach was feasible because of the availability of the recently-established full-scale Associative Transcriptomics (AT) platform comprising 355,536 SNP markers and transcriptome reference comprising 116,098 ordered coding DNA sequence gene models (Havlickova et al., 2018). We could deploy this for a large panel of 288 *B. napus* accessions because of the availability of a recently-developed simple and efficient GSL extraction method (Doheny-Adams et al., 2017).

## 2 Results

### 2.1 Glucosinolates identified in *B. napus* leaves and roots

**Fig. 2:**
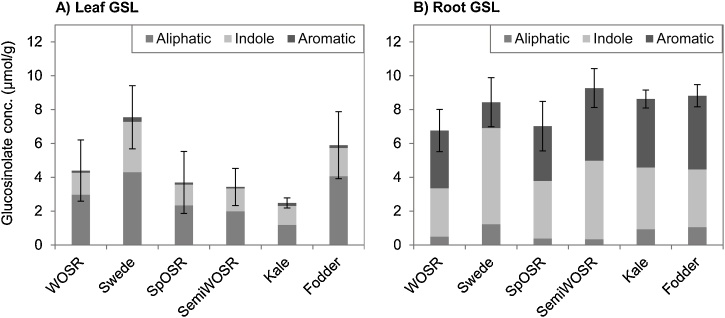
Glucosinolate variations in *B. napus*. Means of glucosinolate (GSL) content in (A) leaf and (B) root of 288 *B. napus* accessions grouped into six crop types. Individual GSLs were grouped according to their structural classes as aliphatic, indole and aromatic GSLs. Abbreviation: spring oilseed rape (SpOSR), semi-winter oilseed rape (SemiWOSR), winter oilseed rape (WOSR), winter fodder (fodder). Error bars represent standard deviations of total GSL.

A subset of 288 diverse *B. napus* accessions with defined crop types of the RIPR panel (Renewable Industrial Products from Rapeseed) (Havlickova et al., 2018) was analyzed for GSL compositions in the leaves and roots of 4-week old plants. Fourteen different GSLs were identified. Out of these, nine are classed as aliphatic (including C_3_, C_4_ and C_5_ types), four indole and one aromatic GSL (Table 1). Detailed profiles are provided in Appendix 1 of Kittipol et al. (2019). To identify relationships between GSL content of leaves and roots, we performed a Spearman’s correlation analysis (Table 2). Within leaf, the total amount of GSL accumulated in the tissue is determined largely by the level of leaf aliphatic GSL (r = 0.91 ***). While both indole and aromatic GSLs are the major GSL classes found in roots, aromatic GSL (i.e. GST) provides a much stronger indication of the total amount of root GSLs (r = 0.75 ***) than root indole GSL (r = 0.41 ***). Significant positive correlations were observed between aliphatic and aromatic GSLs within the same tissue (Leaf: r = 0.62 ***, Root: r = 0.30 ***), as well as between leaf and root (r = 0.50 ***, 0.29 ***), suggest the possibility of co-regulation that is shared between these two classes of GSLs. Whereas, the weak and negative correlations between indole and aromatic GSL within root (r = –0.18 **) and between root and leaf tissues (r = –0.15*, –0.22***) indicate antagonistic relationship between this two GSL classes. Given that different GSL profiles were found between aliphatic-dominated leaf and indole/aromatic-dominated root (Fig. 2), the GSL metabolic pathways between above- and below- ground tissues appears to be regulated differentially yet has some cross-talk between the pathways, which is supported by the weak but significant correlation between total GSLs in the leaf and root (r = 0.28 ***).

**Table 1:** Glucosinolates identified in this study.

Type	Trivial name	Acronym	Systematic R Side chain
**Aliphatic C_3_**	Glucoputranjivin	GJV	1-Methylethyl
**Aliphatic C_4_**	Gluconapin	GNA	3-Butenyl
	Progoitrin	PRO	(2R)-2-Hydroxy-3-butenyl
	Glucoerucin	GER	4-Methylthiobutyl
	Glucoraphanin	GRA	4-Methylsulfinylbutyl
	Glucoraphenin	GRE	4-Methylsulfinyl-3-butenyl
**Aliphatic C_5_**	Glucoalyssin	GAL	5-Methylsulfinylpentryl
	Glucobrassicanapin	GBN	Pent-4-enyl
	Gluconapoleiferin	GNL	2-Hydroxy-pent-4-enyl
**Indole**	Glucobrassicin	GBS	3-Indolylmethyl
	4-Hydroxyglucobrassicin	4-OHGBS	4-Hydroxy-3-indolylmethyl
	4-Methoxyglucobrassicin	4-OMeGBS	4-Methoxy-3-indolylmethyl
	Neoglucobrassicin	neo-GBS	N-Methoxy-3-indolylmethyl
**Aromatic**	Gluconasturtiin	GST	2-Phenethyl

**Table 2:** Spearman’s correlation coefficient analysis of glucosinolate traits.

	TL	L-ali	L-ind	L-aro	TR	R-ali	R-ind	R-aro
Total Leaf (TL)	–							
Leaf Aliphatic (L-ali)	0.91***	–						
Leaf Indole (L-ind)	0.45***	0.14*	–					
Leaf Aromatic (L-aro)	0.62***	0.62***	0.12*	–				
Total Root (TR)	0.28***	0.30***	0.00	0.37***	–			
Root Aliphatic (R-ali)	0.64***	0.68***	0.10	0.50***	0.43***	–		
Root Indole (R-ind)	0.01	–0.10	0.24***	−0.15*	0.41***	−0.04	–	
Root Aromatic (R-aro)	0.18**	0.29***	–0.22***	0.46***	0.75***	0.30***	–0.18**	–
†Total Seed GSL	0.48***	0.54***	0.00	0.40***	0.02	0.43***	–0.20*	0.09

Correlation of mean trait values from 288 accessions of the diversity panel. Significant correlations are indicated.

*** P ≤ 0.001.

** P ≤ 0.01.

* P ≤ 0.05.

† Data for total seed glucosinolates for 151 *B. napus* accessions came from Lu et al. (2014).

### 2.2 Genetic control of leaf glucosinolate variation

Extensive phenotypic variation was observed in leaves for both amount and type of GSLs. The total GSL content ranged from 0.26 to 21.6 μmol/g in leaves, with aliphatic GSLs as the predominant class (64.0% of all leaf GSLs), indole GSLs contributing (32.9%) and a small amount of the aromatic GSL, GST (3.1%). When the *B. napus* diversity panel was assessed by crop type, accessions of the swede crop type were found to contain the greatest amount of GSL (Appendix 3 in Kittipol et al., 2019), with modern winter and spring oilseed rape crop types having the lowest GSL content.

**Fig. 3:**
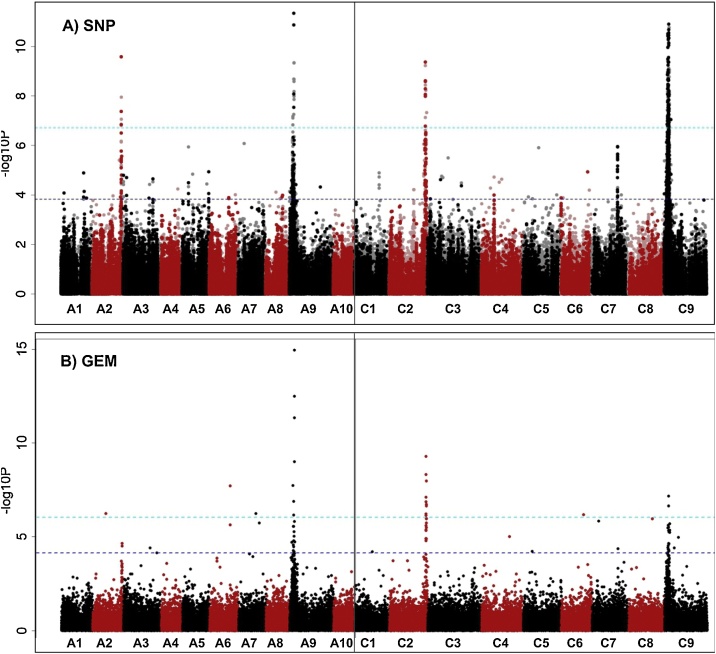
Association analysis for leaf aliphatic glucosinolate content. (A) Manhattan plot showing genome-wide associations for the identification of transcriptome single-nucleotide polymorphism (SNP) markers of 288 *Brassica napus* accessions with leaf glucosinolate content. Marker associations was calculated using a mixed linear model which incorporated population structure and relatedness. The SNP markers are positioned on the x-axis based on the genomic order of the gene models in which the polymorphism was scored. The significance of the trait association, as -log10 P values, plotted on the y-axis. The horizontal purple and cyan lines represent false discovery rate (FDR) threshold at 5% and the threshold for Bonferroni significance of 0.05, respectively. Chromosomes of *B. napus* are labelled A1– A10 and C1 – C9, shown in alternating black and red colors to allow boundaries to be clearly distinguished. Dark opaque points are simple SNP markers (i.e. polymorphisms between resolved bases) and hemi-SNPs that have been directly linkage-mapped, both of which can be assigned to one genome, whereas light points are hemi-SNP markers (i.e. polymorphisms involving multiple bases called at the SNP position in one allele of the polymorphism) for which the genome of the polymorphism cannot be assigned. (B) Association analysis of expression variation-based markers (GEM) with leaf aliphatic glucosinolate. Reads per kb per million aligned reads (RPKM) were regressed against the trait, and R^2^ and P values were calculated for each gene. The gene models are positioned on the x-axis based on their genomic order, with the significance of the associated trait, as -log10 P, plotted on the y-axis. The horizontal purple and cyan lines represent false discovery rate (FDR) threshold at 5% and the threshold for Bonferroni significance of 0.05, respectively (For interpretation of the references to colour in this figure legend, the reader is referred to the web version of this article.).

**Fig. 4:**
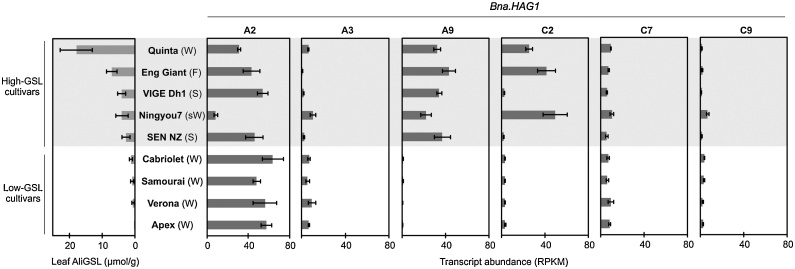
Expression of *Bna.*HAG1 homoeologues in high- and low- leaf aliphatic GSL *B. napus* cultivars. Six orthologues of HAG1 (AT5G61420) are found in *B. napus*, on chromosome A2, A3, A9, C2, C7 and C9. Transcript abundance of *Bna.*HAG1 is expressed as reads per kb per million aligned reads (RPKM), with error bars to indicate standard deviation from four biological replicates of each accessions. Crop type abbreviation: (W), Winter oilseed rape; (F), Winter fodder; (sW), Semiwinter oilseed rape; (S), Swede.

To understand the genetic control of this observed variation we used the established *B. napus* Associative Transcriptomics (AT) platform consisting of 355,536 SNP markers and gene expression matrix with a transcriptome reference of 116,098 ordered coding DNA sequence gene models (Havlickova et al., 2018) to identify molecular marker variation associated with trait variation. As visualized using “Manhattan Plots”, clusters of markers with allelic variation correlated with trait variation indicates regions of the genome containing genes controlling the traits. We undertook AT analysis on all individual GSLs, total GSL and GSLs grouped by type (aliphatic, indole or aromatic). The Manhattan plots are shown in Appendix 5 of Kittipol et al. (2019). As illustrated in Fig. 3, associations for aliphatic GSL content exceeding the Bonferroni-corrected 5% significance threshold with SNP markers were observed in regions of chromosomes A2, A9, C2 and C9. A fifth region exceeding the 5% FDR threshold (but not the Bonferroni-corrected 5% significance threshold) was identified on chromosome C7. These five genomic regions had previously been observed in an AT analysis of total seed GSL content (Lu et al., 2014), suggesting that leaf and seed GSL content are both controlled by the same loci. Investigation of the genes underlying the positions of these five association peaks, as shown in Appendix 9 of Kittipol et al. (2019), revealed at every one an orthologue of HAG1 (AT5G61420), a transcription factor that positively regulated aliphatic GSL biosynthesis. In addition, of the associations between Gene Expression Markers (GEM) and leaf aliphatic GSL content that exceeding the 5% FDR threshold six were detected for genes involved directly in aliphatic GSL biosynthesis (Appendix 11 in Kittipol et al., 2019). Two of these genes are known to be involved in the aliphatic amino acid chain elongation, an orthologue of AT5G23020, a methythioalkymalate synthase (MAM3) was found on A3 and an orthologue of AT5G23010, MAM1, on C7. Two genes involved in the core GSL structure biosynthesis, an orthologue of AT1G16410, a cytochrome P450 CYP79F1 and an orthologue of AT1G78370, a glutathione S-transferase TAU 20 (GSTU20) were identified on chromosome C5 and A7 respectively. Two orthologues of HAG1, *Bna.*HAG1*.A9* and *Bna.*HAG1*.C2*, were also identified amongst the top GEMs, implicating the transcript abundance levels of these genes in the control of aliphatic GSL in the leaf. To test this, we analyzed leaf transcript abundance on four biological replicates for all six HAG1 orthologues in 5 high leaf GSL and 4 low leaf GSL *B. napus* accessions. Consistent with the AT results, as shown in Fig. 4, expression of *Bna.*HAG1*.A9* and *Bna.*HAG1*.C2* showed strong positive correlation with level of aliphatic GSL in leaves, whereas the orthologues on A3, C7 and C9 were expressed at relatively low levels. The remaining orthologue, on chromosome A2 was relatively highly expressed in all accessions so this copy appears to be either encode a non-functional protein or has lost its role in the control of leaf glucosinolate biosynthesis by subfunctionalization.

### 2.3 Genetic control of root glucosinolate variation

Extensive phenotypic variation was observed in roots for both amount and type of GSLs. The total GSL content in roots ranged from 2.4 to 17.1 μmol/g (Appendix 1 in Kittipol et al., 2019). In contrast to leaves, indole GSLs (47.7%) and the aromatic GSL GST (45.0%) formed the major classes, with aliphatic GSLs being a minor component (7.3%).

**Fig. 5:**
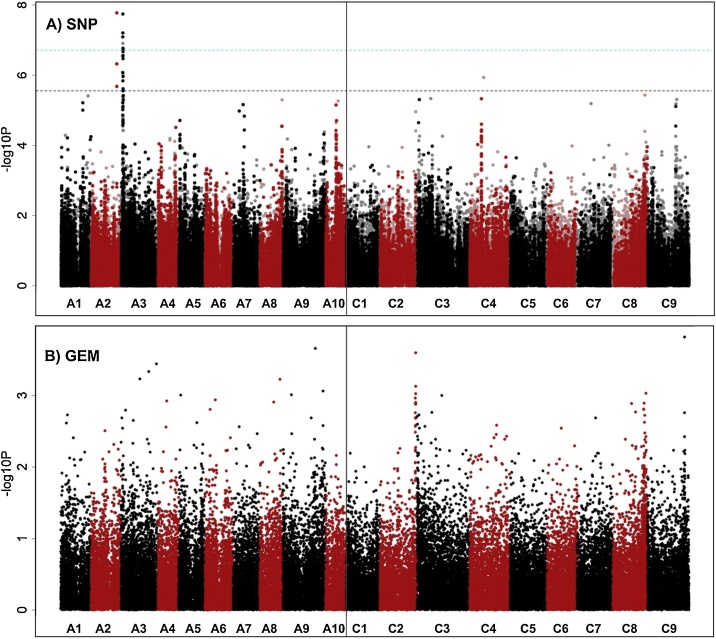
Association analysis for root aromatic glucosinolate content. (A) Manhattan plot showing genome-wide associations for the identification of transcriptome single-nucleotide polymorphism (SNP) markers of 288 *Brassica napus* accessions with leaf glucosinolate content. Marker associations was calculated using a mixed linear model which incorporated population structure and relatedness. The SNP markers are positioned on the x-axis based on the genomic order of the gene models in which the polymorphism was scored. The significance of the trait association, as -log10 P values, plotted on the y-axis. The horizontal purple and cyan lines represent false discovery rate (FDR) threshold at 5% and the threshold for Bonferroni significance of 0.05, respectively. Chromosomes of *B. napus* are labelled A1– A10 and C1 – C9, shown in alternating black and red colors to allow boundaries to be clearly distinguished. Dark opaque points are simple SNP markers (i.e. polymorphisms between resolved bases) and hemi-SNPs that have been directly linkage-mapped, both of which can be assigned to one genome, whereas light points are hemi-SNP markers (i.e. polymorphisms involving multiple bases called at the SNP position in one allele of the polymorphism) for which the genome of the polymorphism cannot be assigned. (B) Association analysis of expression variation-based markers (GEM) with leaf aliphatic glucosinolate. Reads per kb per million aligned reads (RPKM) were regressed against the trait, and R^2^ and P values were calculated for each gene. The gene models are positioned on the x-axis based on their genomic order, with the significance of the associated trait, as -log10 P, plotted on the y-axis (For interpretation of the references to colour in this figure legend, the reader is referred to the web version of this article.).

To identify loci controlling the level and composition of GSLs in roots, we undertook AT analysis on all individual GSLs, total GSL and GSLs grouped by type (aliphatic, indole or aromatic). The Manhattan plots are shown in Appendix 4 of Kittipol et al. (2019). For the root aliphatic GSLs, SNP associations revealed the same controlling loci on A2/C2 and A9/C9 as in leaves, and furthermore *Bna.HAG1.A9* is also identified as one of the top GEMs (*p* =  2.10×10^−9^). For the aromatic GSL (i.e. GST), an exceptionally well-defined association peak, with SNP markers exceeding the Bonferroni-corrected 5% significance threshold, was identified on chromosome A3, as shown in Fig. 5. The genes in this region as listed in Appendix 14 of Kittipol et al. (2019) include an orthologue of HAG3 (AT5G07690), a transcription factor shown (from studies in *A. thaliana*) to regulate aliphatic GSL biosynthesis. The expression level of *Bna.*HAG3*.A3* in the AT platform dataset of Havlickova et al (2018) is low across all accessions. The functional genotypes had been derived from re-sequencing of leaf transcriptome, so GEMs would not be identifiable for genes with root-specific expression patterns. We therefore performed differential expression analyses based on root transcriptome re-sequencing of 4 accessions with high root aromatic GSLs and 4 accessions with low root aromatic GSLs, as listed in Appendix 15 of Kittipol et al. (2019), each with 4 biological replicates. *Bna.*HAG3*.A3* expression was found to be highly correlated with aromatic GSL content (log_2_ fold-change = 14.8; p = 5.47 × 10^-11^) with expression of *Bna.*HAG3*.A3* high in high-root aromatic GSL group and very low in the low-root aromatic group, as shown in Supplementary Figure S1, confirming *Bna.*HAG3*.A3* as an excellent candidate for controlling this trait.

In order to identify differential expression of genes that might be regulated by *Bna.HAG3.A3* in such a way as to limit potential confounding effect between GSL pathways, we performed a stringent root differential expression analysis (log_2_ fold-change ≥ 4; p ≤ 1 × 10^−10^) between accessions N01D-1330 and KARAT, which differ in root aromatic GSLs but both of which are low in aliphatic GSLs. This analysis revealed 107 genes with BLAST hits to annotated *A. thaliana* genes, including an orthologue of MAM3 (AT5G23020) on chromosome A3, an orthologue of *IMPI2* (AT2G43100) on chromosome C4 and orthologues of *CYP83A1* (AT4G13770) on each of chromosomes A4 and C4, as shown in Appendix 16 of Kittipol et al. (2019). All of these show higher expression in the high root aromatic GSL accession. In *Arabidopsis*, MAM3 was identified as the key enzyme catalyzing chain elongation of methionine-derived GSLs (Textor et al., 2007) and CYP83A1 can oxidize both aliphatic and aromatic aldoximes (Naur et al., 2003). In *B. napus*, we found that the expression of *Bna.*MAM3*.A3*, *Bna.CYP83A1.A4* and *Bna.CYP83A1.C4* all had significant positive correlations with aromatic GSL in roots (Appendix 17 in Kittipol et al., 2019). GST is a derivative of the chain-elongated homophenylalanine but the genes involved in the chain-elongation of phenylalanine of aromatic GSL pathway are unclear. This result suggests that *Bna.*MAM3*.A3* may play an important role in phenylalanine elongation for aromatic GSL biosynthesis in *B. napus*.

### 2.4 Relationships between glucosinolate content of vegetative tissues and seeds

In order to understand the relationship of GSLs between vegetative tissues and seeds, we added the seed GSL data from Lu et al. (2014) to the leaf and root data collected from this study and extended the Spearman’s correlation analysis shown in Table 2 to include seeds. Aliphatic GSL exhibited the strongest correlations between organs, in particular between leaf and the other two organs (Leaf-Root: r = 0.68 ***, Leaf-Seed: 0.54 ***, Seed-Root: 0.43 ***). These significant positive correlations indicate that natural variation observed in aliphatic GSL between the organs could be regulated by long-distance transport or a master regulator of the aliphatic biosynthetic pathway that controls the biosynthesis of aliphatic GSLs in all of these organs. To investigate whether variation in transport or biosynthesis processes explained the natural variations in aliphatic GSL pattern between leaf and seed *B. napus*, we analyzed additional seed data for associations with the orthologues of *Arabidopsis* GSL transporters, GTR1 (AT3G47960) and GTR2 (AT5G62680). In *B. napus* genome, four orthologues of GTR1 (on C3 and A6) and five orthologues of GTR2 (on C3, C9, A6 and A9) were found but none of the copies showed associations with seed, leaf or root aliphatic GSL. Although *Bna.*GTR2*.A9* and *Bna.*GTR2*.C9* were found in parts of the genome within the SNP and GEM association peaks on chromosome A9 and C9, no correlation between gene expressions and aliphatic contents was observed across the tissues (Appendix 18 in Kittipol et al., 2019). Comparison of the AT plots for total seed GSL, leaf aliphatic and root aliphatic GSLs showed that they all shared four common association peaks on chromosome A2, A9, C2 and C9 which correspond to the HAG1 orthologue-containing control loci. Furthermore, comparison of aliphatic GSLs in leaf and seed, as shown in Supplementary Figure S2 revealed two distinct classes: one with relatively high GSL in both organs and one with relatively low GSL in both organs. The lack of any accession with high GSL in the leaves and low GSL in the seeds indicates the basis of aliphatic GSL variations between plant tissues to be from the amount synthesized, as controlled by orthologues of HAG1, and not by variation in the transport processes.

## 3 Discussion

### 3.1 Aliphatic glucosinolates

The Polish spring rape cultivar Bronowski is known to be the genetic source for this trait deployed in all commercial low-seed GSL *B. napus* cultivars through selective breeding (Rosa et al., 1997). This reduction in oilseed GSLs is due to reduction in aliphatic GSLs (Kondra and Stefansson, 1970; Rucker and Rudloff, 1991). However, the molecular mechanism underlying the low seed GSL trait in oilseed rape was unclear. Some studies reported no significant correlation between seed and leaf GSL in *B. napus* canola cultivars (Porter et al., 1991; Fieldsen and Milford, 1994), leading to an assumption that inhibition of the GSL transport processes could have given rise to the low-seed GSL trait in *B. napus*. This hypothesis was supported by the report on the two nitrate/peptide transporter family, GTR1 and GTR2, controlling GSL accumulation in *A. thaliana* seeds (Nour-Eldin et al., 2012). Although orthologues of GTR2 are found in close proximity to causative loci controlling low-seed GSL trait in *B. napus* (Lu et al., 2014), we identified no accession with low seed GSL and the high leaf GSL that would be expected from blocking transport from the leaf, as was observed in *A. thaliana*. Neither did we identify SNP or GEM associations between GTR1 or GTR2 orthologues and GSL traits. Instead, our data reveals significant positive correlation between seed and leaf GSLs where seed GSL profile is a good reflection of the profile found in the leaf (Table 2, Supplementary Fig. S2 and Appendix 19 in Kittipol et al., 2019). Previous work in *A. thaliana* has shown a similar positive correlation with the level of aliphatic GSLs in the leaves representing the minimal concentration of aliphatic seed GSL assuming there were no variation in GSL transport from the leaves to the seeds (Kliebenstein et al., 2001b). Aliphatic GSLs predominate in *B. napus* leaf and seed, so it is not surprising that the same gene associations were detected for total seed GSL (Harper et al., 2012) and total leaf GSL (Appendix 5 & Appendix 10 in Kittipol et al., 2019). Genetic variation for the reduced GSL level in seed, which reflected in the reduced GSL level in leaf, was due to structural changes in the region of *B. napus* genome containing the key regulator of aliphatic GSL biosynthetic genes as a result of breeding-directed selection. Our gene expression analyses confirm the results, i.e. that low-leaf aliphatic GSL lines such as ‘Cabriolet’ and ‘Apex’, have non-functional HAG1 orthologues on chromosomes A9 and C2 in place of functional genes in high aliphatic GSL lines (Appendix 12 & Appendix 13 in Kittipol et al., 2019). Our results are consistent with the genome sequence of the low-GSL cultivar Darmor-*bzh*, in which orthologues of HAG1 have been lost on chromosome A9 and C2 but no sequence changes in GTR1 and GTR2 orthologues were identified (Chalhoub et al., 2014).

### 3.2 Aromatic glucosinolates

Although homophenylalanine-derived GSL is prevalent in *B. napus* roots, few ecotypes of *A. thaliana* produce this class of GSL, and then in very small amounts (Brown et al., 2003). The resulting inability to use the model plant *A. thaliana* to study aromatic GSL and the challenges of working with *B. napus* complex polyploidy has limited the advancement in the understanding of the aromatic biosynthetic pathway. To overcome these challenges, we combined AT with a differential gene expression analysis in root tissues. The region of chromosome A3 showing strong association with variation in root aromatic GSL (Fig. 5) contained an orthologue of HAG3 (Appendix 14 in Kittipol et al., 2019). Compared with other orthologues, *Bna.*HAG3*.A3* contained the highest frequency of polymorphisms, particularly SNPs, which showed strong association with variation in GST content of roots. Using the expression data from root RNA-seq, we have found higher expressions of *Bna.*HAG3*.A3* gene in high-root aromatic GSL lines and lower expression in low root aromatic GSL lines, supporting our hypothesis. Our interpretation is that *Bna.*HAG3*.A3*, an orthologue of a known regulator of aliphatic GSL in *A. thailana*, is a key regulator of root aromatic GSL biosynthesis in *B. napus*. Furthermore, our results indicate that *Bna.*HAG3.*A3* regulates a biosynthetic pathways shared between aliphatic and aromatic GSLs. Through differential expression analysis we identified *Bna.*MAM3.*A3* amongst the genes with largest changes in their expression between accessions (Appendix 16 in Kittipol et al., 2019). Roots of *B. napus* are dominated by a chain-elongated homophenylalanine aromatic GSL, GST, but genes involved in the chain-elongation of phenylalanine are unknown. We propose that *Bna.*MAM3*.A3,* previously known to be part of aliphatic pathway, is also involved in the chain-elongation of phenylalanine in *B. napus*. Consistent with this hypothesis is the observation that MAM3 has a broad substrate specificity in addition to methionine-derived 2-oxoacids where MAM3 is able to form condensation reaction with phenylpyruvate leading to GST production (Textor et al., 2007). Quantitative Trait Locus mapping studies in *A. thaliana* for aromatic GSL reported *GS-Elong* locus (comprising MAM1, MAM2 and MAM3), which controls total leaf aliphatic GSL, to also be the major QTL for controlling phenylalanine elongation (Kliebenstein et al., 2001a). This is also consistent with our hypothesis that chain elongation of methionine-derived aliphatic GSLs and phenylalanine-derived aromatic GSLs share a pathway.

## 4 Conclusions

Glucosinolate profiles in *B. napus* accessions differ extensively in both type and amount. Aliphatic GSL content in seeds and roots reflect those in leaves and is regulated by *Bna.HAG1*.A9 and *Bna.HAG1*.C2. Aromatic GSLs predominate in the root and we implicate *Bna.HAG3*.*A3* in their control. There are implications for the manipulation of GSLs for modulation of interactions between the important crops of this species and various pests and diseases. Firstly, blockage of glucosinolate transport into seeds (thus achieving the low seed GSL content needed for oilseed rape quality whilst maintaining high aliphatic GSL content in vegetative tissues) has not yet been achieved in the available germplasm and represents an opportunity to be explored. Secondly, there is a simple genetic basis for the variation observed for root aromatic GSL content and impacts of this variation on below-ground interactions can now be explored.

## 5 Materials and methods

### 5.1 Growth of plant material for glucosinolate content

*Brassica napus* (Oilseed rape) leaves and roots from 288 genotypes of the Renewable Industrial Products from Rapeseed (RIPR) diversity population (Havlickova et al., 2018) were harvested for GSL extraction four weeks after sowing, as described in detail in Kittipol et al. (2019). Four biological replicates of each accessions were grown. At harvest, leaf and root samples were wrapped in labelled foil and immediately frozen in liquid nitrogen. There are 56 Modern Winter oilseed rape (OSR), 65 Winter OSR, 6 Winter Fodder, 121 Spring OSR, 26 Swede and 14 Exotic varieties within this panel (Appendix 1 in Kittipol et al., 2019).

### 5.2 Glucosinolate quantification

A complete description of the GSL extraction methodology and analysis is presented in Kittipol et al. (2019) and Doheny-Adams et al. (2017). Briefly, GSL mixture from freeze-dried ground leaves or roots were extracted with 80% methanol (v/v), purified and desulfated overnight (Kittipol et al., 2019). Glucotropaeolin was added as an internal standard prior to extraction. Desulfoglucosinolates (dsGSL) were separated by HPLC coupled with photodiode array detector using reverse phase C18 column (5μ ODS(2), 150 mm × 4.6 mm) at 30 °C with mobile phase solutions consisting of 100% diH_2_O and 30% (v/v) acetonitrile, as described in Doheny-Adams et al (2017).

### 5.3 Statistical analysis

Statistical analyses were carried out with R statistical software (R core team, 2013). Spearman’s correlation analysis was used to analyze the relationship between different groups of GSL in different organs (Table 2). Spearman’s correlation was an appropriate type of correlation coefficient because it is more robust to work with the large variabilities and skewed distribution of the levels of GSLs.

### 5.4 Associative transcriptomics

Functional genotype was constructed (Havlickova et al., 2018) by mapping leaf RNA-sequence data onto the reference sequence of ordered Brassica A and C genome-based pan-transcriptomes (He et al., 2015), using the method described in (Bancroft et al., 2011). To reduce errors in SNP identification and assessment of linkage disequilibrium, filtering and quality checking parameters were applied as described in (Havlickova et al., 2018), producing a set of 355 536 SNP markers, of which 256 397 SNP had a minor allele frequency (MAF) > 0.01. Transcript abundance was quantified and normalized as reads per kb per million aligned reads (RPKM) for each sample and 53 889 CDS models was detected with significant expression (> 0.4 RPKM). Full detail of the methods is described in Kittipol et al. (2019).

The statistical software R was used to perform Associative Transcriptomics was performed using R, as detailed in Kittipol et al. (2019) and Havlickova et al. (2018). SNP-based analyses were performed with Genome Association and Prediction Integrated Tool (GAPIT) R package using mixed linear model that includes both fixed and random effects. SNP markers are positioned on the x-axis based on the genomic order of the CDS gene model in which the polymorphism was scored. The significance of the trait association, as –log_10_P values, was plotted on the y-axis. For GEM-based analyses, fixed-effect linear model was calculated in R software, with trait score as the response variable and RPKM values plus the Q matrix inferred by PSIKO as explanatory variables. False discovery rate (FDR) (Benjamini and Hochberg, 1995) and threshold for Bonferroni (Dunn, 1961) corrections were used to set significance threshold at P < 0.05.

### 5.5 Differential expression analysis of root RNA-seq data

Differential gene expression was analyzed using root transcriptome sequences from four biological replicates. The methods in Bioconductor package EdgeR (Robinson et al., 2009) were used to identify differentially expressed genes, as described in Kittipol et al. (2019).

### 5.6 Accession numbers

Short read sequence data have been deposited at the Sequence Read Archive under BioProject ID: PRJNA524101

## Author contributions

V.K. designed and performed the experiments, analyzed the data and wrote the manuscript.

Z.H. performed root differential expression analysis.

L.W. grown plant material and extracted roots RNA for the differential expression experiment.

T.D-A. helped in method development for glucosinolate quantification and performed some HPLC analysis. S.L. performed some HPLC analysis.

I.B. designed experiments, interpreted results and edited the manuscript.

## Funding

This work was supported by UK Biotechnology and Biological Sciences Research Council [grant number BB/L002124/1]. We thank the High-Throughput Genomics Group at the Wellcome Trust Centre for Human Genetics (funded by Wellcome Trust grant reference 090532/Z/09/Z) for the generation of mRNAseq and genomic sequencing data. VK received support from Radhika V Sreedhar Scholarship Fund from the Department of Biology, University of York and Scholarships for Overseas Students from the University of York.

## One sentence summary

In *Brassica napus*, orthologues of *HAG1* control variation of aliphatic glucosinolates in leaves and orthologues of *HAG3* control variation of aromatic glucosinolates in roots.
