# Supplementary material for: Genetic architecture of glucosinolate variation in Brassica napus
Source: J Plant Physiol. 2019 Sep;240:152988. doi: 10.1016/j.jplph.2019.06.001 (PMC6739596; doi:10.1016/j.jplph.2019.06.001)
Supplement: Supplementary file 1 [file mmc1.pdf]

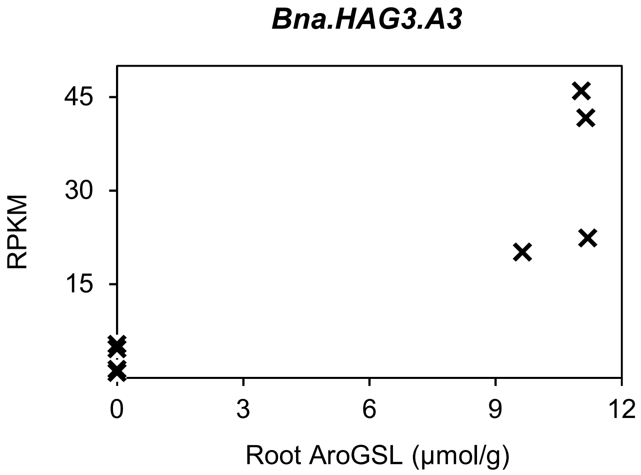

Figure S1. ***Bna.HAG3.A3*** **root expression against root aromatic GSL levels.** Means of four biological replicates of 8 *B. napus* accessions are shown.

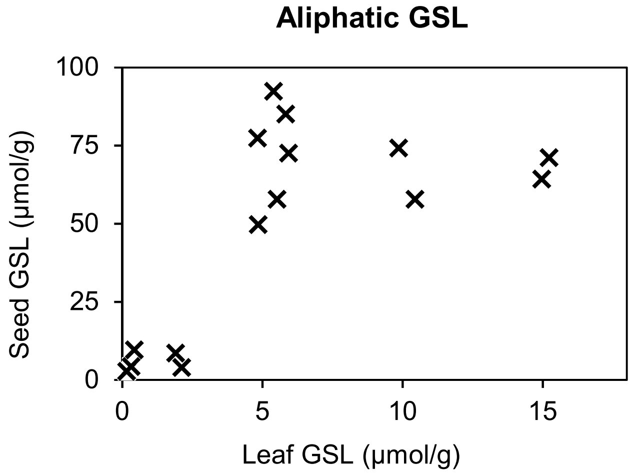

Figure S2. Relationship of aliphatic glucosinolates between leaf and seed tissues. Mean of four biological replicates of 15 *B. napus* accessions are shown.
